# Chemopreventive Effects of Sarcophine-diol on Ultraviolet B-induced Skin Tumor Development in SKH-1 Hairless Mice

**DOI:** 10.3390/md7020153

**Published:** 2009-04-30

**Authors:** Xiaoying Zhang, Ajay Bommareddy, Wei Chen, Michael B. Hildreth, Radhey S. Kaushik, David Zeman, Sherief Khalifa, Hesham Fahmy, Chandradhar Dwivedi

**Affiliations:** 1 Department of Pharmaceutical Sciences, South Dakota State University, Brookings, SD 57007, USA; E-Mails: violett_chang@hotmail.com; vigo688@hotmail.com; 2 Department of Pharmacology & Chemical Biology and University of Pittsburgh Cancer Institute, Pittsburgh, PA 15213, USA; E-Mail: Ajay.bommareddy@gmail.com; 3 Department of Veterinary Science, South Dakota State University, Brookings, SD 57007, USA; E-Mail: david.zeman@sdstate.edu; 4 Department of Biology/Microbiology, South Dakota State University, Brookings, SD 57007, USA; E-Mails: Michael.Hildreth@sdstate.edu; Radhey.Kaushik@sdstate.edu; 5 Faculty of Pharmacy, Misr International University, Cairo, Egypt; E-Mail: jkhalifa_99@yahoo.com

**Keywords:** Chemopreventive agent, Sarcophine-diol, Apoptosis, Skin cancer

## Abstract

Sarcophine-diol (SD), one of the structural modifications of sarcophine, has shown chemopreventive effects on 12-dimethylbenz(a)anthracene-initiated and 12-*O*-tetradecanoylphorbol-13-acetate-promoted skin tumor development in female CD-1 mice. The objective of this study was to determine the chemopreventive effects of SD on UVB-induced skin tumor development in hairless SKH-1 mice, a model more relevant to human skin cancer, and to determine the possible mechanisms of action. Carcinogenesis was initiated and promoted by UVB radiation. Female hairless SKH-1 mice were divided into two groups having 27 mice in each group: control and SD treatment. The control group was topically treated with 100 μL acetone and SD treatment group was topically treated with SD (30 μg/100 μL in acetone) 1 hour before each UVB radiation for 32 weeks. Tumor counts were recorded on a weekly basis for 30 weeks. Effects of SD on the expression of caspases were investigated to elucidate the possible mechanism of action. The proteins from epidermal homogenates of experimental mice were used for SDS-PAGE and Western blotting using specific antibodies against caspase-3, caspase-8 and caspase-9 respectively. TUNEL assay was used for determining DNA fragmented apoptotic cells *in situ*. Results showed that at the end of experiment, tumor multiplicity in control and SD treatment groups was 25.8 and 16.5 tumors per mouse respectively. Furthermore, Topical treatment of SD induced DNA fragmented apoptotic cells by upgrading the expressions of cleaved caspase-3 and caspase-8. This study clearly suggested that SD could be an effective chemopreventive agent for UVB-induced skin cancer by inducing caspase dependent apoptosis.

## 1. Introduction

Non-melanoma skin cancer (NMSC), including basal cell carcinoma (BCC) and squamous cell carcinoma (SCC) are the most common malignant neoplasms in human [1]. It has been estimated that more than one million cases of BCC and SCC are diagnosed each year in the US alone [2], which is equivalent to the incidence of malignancies in all other organs combined [3,4].

Chronic exposure to ultraviolet (UV) radiation is responsible for about 90% of human NMSCs [5]. Over-exposure to UV from the sun can cause sunburn, skin damage and ultimately, skin cancer. Solar UV radiation is conventionally divided into UVA (320–400 nm), UVB (280–320 nm) and UVC (200–280 nm). The UV light that reaches the earth is composed of about 90% UVA and 10% UVB; whereas, UVC does not penetrate the earth’s atmosphere [6]. In addition, the level of UVB reaching the earth’s surface is controlled largely by the amount of ozone in the atmosphere [7,8]. Both UVA and UVB are being studied for their skin cancer-causing potential, but currently UVB is thought to be the most important etiologic factor. Thus, UVB is the most frequently used photocarcinogen in animal studies [9,10]. UVB can act in mouse skin models as a complete carcinogen, meaning that UVB can function as an initiator as well as a promoter [11,12].

**Figure 1:**
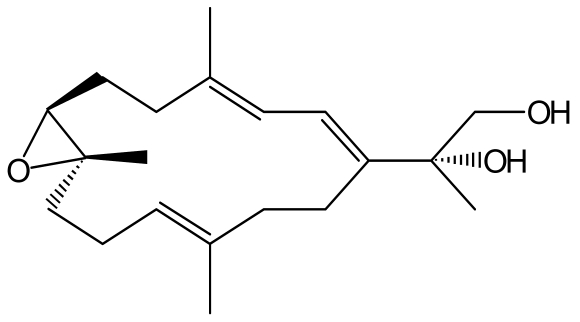
The structure of sarcophine-diol (SD).

These observations suggest that developing effective approaches and novel strategies to prevent UVB-caused human NMSC is imperative to the society. Recently, there has been a considerable interest in the use of marine natural products for the chemopreventive activity against skin tumor development [13–18]. Our laboratory has reported that semisynthesis of sarcophine derivatives such as sarcophine-diol (SD, Figure 1) showed high chemopreventive effects against 12-dimethylbenz(a)anthracene (DMBA)-initiated and 12-*O*-tetradecanoylphorbol-13-acetate (TPA)-promoted skin carcinogenesis [17]. The objective of this study was to determine the chemopreventive effects of SD on UVB-induced skin tumor development in female SKH-1 hairless mice, an experimental model more relevant to human skin cancer development. Tumorigenesis is associated with loss of apoptotic death of cells [19]. Accordingly, in the present study, effects of SD treatment on apoptosis after UVB exposure to SKH-1 mice were determined to elucidate the possible mechanisms of action of SD in the UVB-mouse model.

## 2. Results

### SD treatment did not affect body weight gain of SKH-1 mice

There was no significant difference in weight gain between control and SD treatment groups throughout the entire experiment (data are not shown). SD pre-treatment did not show any apparent skin toxicity to SKH-1 mice and also did not influence the normal growth and development of the mice during the whole experimental period.

### SD treatment did not significantly inhibit the incidence of skin tumors in SKH-1 mice

**Figure 2:**
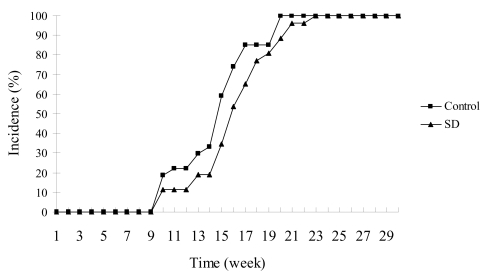
Effects of SD treatment on tumor incidence in SKH-1 mice. Skin tumors appeared in the 10^th^ week of UVB-promotion phase in both control and SD treatment groups. SD pre-treatment did not significantly (*P* < 0.05) decrease the appearance of tumors throughout the experiment.

The effects of SD treatment on the incidence of skin tumors in SKH-1 mice are shown in Figure 2. Skin tumors appeared in the 10^th^ week of UVB-promotion phase in both control and SD treatment groups. Skin tumor incidence was 100% in both control and SD treatment groups respectively by 23 weeks of UVB-promotion. Results showed that SD pre-treatment did not have significant (*P* < 0.05) effect on the incidence of tumor development throughout the experiment.

### SD treatment inhibited tumor multiplicity in SKH-1 mice

**Figure 3:**
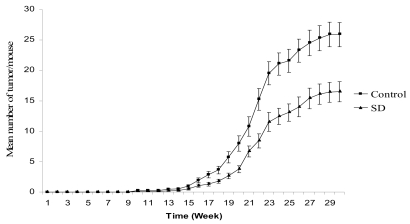
Effects of SD treatment on tumor multiplicity in SKH-1 mice. SD pre-treatment significantly (*P* < 0.05) decreased tumor multiplicity from 15^th^ week to 30^th^ week of UVB-promotion phase. Each point represents mean number of tumor per mice ± SE derived from 27 mice.

The effects of SD treatment on tumor multiplicity are shown in Figure 3. The mean number of tumors per mouse was 25.8 and 16.5 in control and SD treatment groups respectively accounting for 36% inhibition in SD treated group at the end of the experiment. Overall, SD pre-treatment resulted in a significant (*P* < 0.05) reduction in tumor multiplicity from 15^th^ week in the promotion phase to the end of experiment (the 30^th^ week of UVB-promotion).

### SD treatment inhibited the percent of total tumor area to total back area of SKH-1 mice

**Figure 4:**
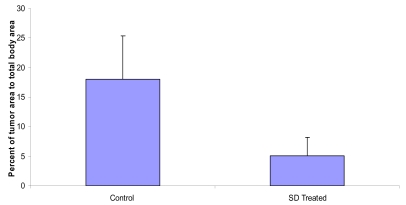
Effects of SD treatment on tumor area in SKH-1 mice. Average ratio of total tumor area to total back area of the SKH-1 mice.

The effects of SD treatment on tumor area are presented in Figure 4. As shown in Figure 4, the mean ratio of total tumor area to total back area was 18.0% and 5.0% in control and SD treatment groups respectively accounting for 73% inhibition in tumor area in SD treated group at the end of the experiment (*P* < 0.05).

### UVB radiation induced squamous cell carcinoma in SKH-1 mice

The histopathological examination of tumor progression was investigated after 30 weeks of the promotion. Results suggested that both control and SD treatment group were showing squamous cell carcinoma in the skin (pictures not shown).

### SD treatment induced caspase-3, -8 expressions but not caspase-9 in SKH-1 mice

**Figure 5:**
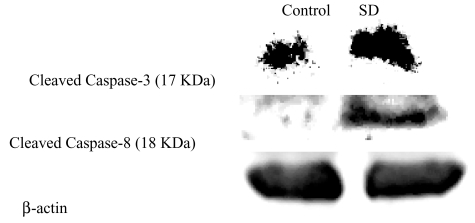
Effects of SD treatment on expressions of cleaved caspase-3 and caspase-8 in SKH-1 mice. At the end of protocol for mechanistic studies as mentioned in materials and methods, proteins were isolated from epidermal tissues of mice; lysates were prepared and subjected to Western blot analysis to determine the expression of different proteins. β-actin was used to verify equal loading of the samples for each membrane.

Epidermal lysates were collected from mice of both control and SD treatment group with 19 weeks of UVB promotion (shortened protocol), when first tumor appeared in both groups. The effects of SD treatment on caspase-3 and caspase-8 expressions are shown in Figure 5. According to the density of bands, SD treatment increased 1.5 times of the expression of cleaved caspase-3 at 17 KDa. Moreover, as shown in Figure 5, the cleaved caspase-8 band at 18 KDa for control group is very faint and can be hardly observed, whereas, the band for SD Treated group is very intense and dark. However, the bands for cleaved caspase-9 in both control and SD treated group were not observed meaning that SD treatment did not increase the expression of caspase-9 (data not shown).

### SD treatment increased DNA fragmented apoptotic cells in SKH-1 mice

**Figure 6:**
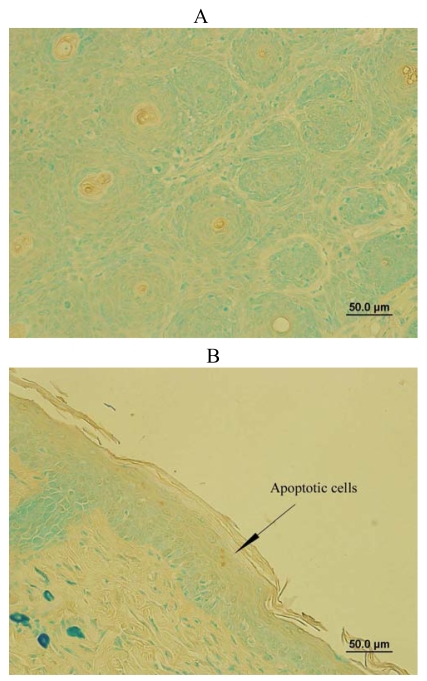
Effects of SD treatment on DNA fragmentation of cells. The skin samples from mechanistic studies were used to determine DNA fragmented cells in situ. Normal cells show green color and apoptotic cells with DNA fragmentation display brown color nuclear staining as showed by an arrow. (A) The image from a control mouse skin and (B) The image from a SD treated mouse skin mainly represent four independent observations from two mouse skins respectively.

DNA fragmentation is the biochemical hallmark of apoptosis, an irreversible event that commits the cell to die [22,23]. TUNEL staining was used to localize apoptotic cells with DNA fragmentation in the mouse skin samples from mechanistic study protocol *in situ*. As shown in Figure 6A, all the cells from control group expressed green color, which were normal cells without DNA fragmentation. However, SD treatment for 19 weeks of the promotion phase modestly increased some DNA fragmented apoptotic cells with brown color nuclear staining as shown in Figure 6B. Thus, SD treatment induced DNA fragmentation of cells in SKH-1 mice.

## 3. Discussion

In recent years, marine natural products such as sarcophytol A, a cembranoid isolated from the Okinawan soft coral *Sarcophyton*, have gained considerable attention as cancer chemopreventive agents [13–18], Sarcophytol A was studied by the National Cancer Institute at a preclinical trial level for skin cancer [14]. However, the major limitation with sarcophytol A is its supply, since it is available only in minute quantities in the soft coral [24].

Sarcophine, a fish toxin that acts as the chemical defense system against natural predators by inhibiting various vital enzymes such as cholinesterase and phosphofructokinase, is one of the most abundant cembranolides, also isolated from the Red Sea *Sarcophyton glaucum* with yields up to 3% of animal dry weight [14,15,20,25]. SD and sarophine-triol (ST) are two structural modifications of sarcophine. Studies showed that SD and ST were superior to Sarcophytol A in inhibiting Epstein-Barr virus early antigen (EBV-E) activation induced by TPA [15]. Moreover, our laboratory has reported that SD and ST show chemopreventive effects against skin carcinogenesis in mice [16–18]. For example, ST has been shown to have chemopreventive effects on both chemical- and UVB-induced skin tumor development in mice by inducing expressions of caspases [16,18]; SD has showed chemopreventive effects on DMBA-initiated and TPA-promoted skin tumor development in female CD-1 mice [17]. However, chemopreventive effects of SD have not been investigated on UVB-induced skin tumor model, an experimental model more relevant to human skin cancer development.

In the present study, for the first time we provide clear evidence that topical application of SD (30 μg/application) has modest chemopreventive effects against UVB-induced skin tumor development in female SKH-1 mice. SD treatment significantly (*P* < 0.05) decreased tumor multiplicity (36% inhibition) and the percent of tumor area in mice (72% inhibition). Moreover, SD pre-treatment inhibited UVB-induced skin tumor development in mice at a very low concentration (30 μg per application) as compared to other reported chemopreventive agents which resulted in similar effects at milligram applications [21, 26–29]. For example, topical application of α-santalol inhibited UVB-induced skin tumor development in mice at 5 mg per application [21,26]; topical application of (−)-epigallocatechin-3-gallate (EGCG) at 1 mg/cm^2^ skin area per application prevented photo-carcinogenesis in wild-type (C3H/HeN) mice [27]; topical application of silymarin protect photocarcinogenesis in SKH-1 mice at 9 mg per application [28] and silibinin at 9 mg per application prevented UV radiation-caused skin damages in SKH-1 hairless mice [29].

Loss of apoptotic cell death is one of the responsible for tumorigenesis [19]. Apoptosis, a programmed cell death, is carried out by a family of cysteine proteases which are caspases [30]. In a classical apoptotic cascade, there are two pathways for apoptosis: extrinsic pathway is related with the cleavage of caspase-8 and intrinsic pathway which is mediated by the activation of caspase-9, both of which activate caspase-3. Activation of caspase-3 results in the cleavage of the inhibitor of the caspase-activated deoxyribonuclease (ICAD) and the caspase-activated deoxyribonuclease (CAD) becomes active leading to DNA fragmentation and apoptotic cell death [31,32].

Based on these observations, epidermal lysates of mice from the mechanistic study protocol were extracted to assess whether SD could inhibit tumorigenesis in UVB-model by inducing apoptosis. Consistent with these notions, the results suggest that SD pre-treatment increased the expressions of caspase-3 and caspase-8, and DNA fragmented cells as compared to control. However, SD did not increase the cleavage of caspase-9. These findings are consistence with our previous studies in chemical-induced skin cancer mouse model [17]. Therefore, SD treatment in both models may induce apoptosis through extrinsic pathway rather than intrinsic pathway. When compared with ST which contains one additional hydroxyl group, ST increased the expression of caspase-9 in both chemical- and UVB-induced skin tumor mouse models [16,18]. Although, both ST and SD are structural modifications of sarcophine and differ from each other just by one hydroxyl group, their mechanisms of action are not similar in both models.

Our previous *in vitro* studies also investigated the mechanisms of action of SD in the human epidermoid carcinoma A431 cell line [33]. The results showed that SD treatment led to a concentration-dependent decrease in cell viability and cell proliferation in human epidermoid carcinoma A431 cells as assessed by MTT and BrdU incorporation assays. Moreover, SD treatment induced a strong apoptosis and significantly (*P* < 0.05) increased DNA fragmentation by upgrading the activity and expression of caspase-3 through activation of upstream caspase-8 in A431 cells. These findings are consistent with the results of the current investigation.

## 4. Experimental Section

### Materials and reagents

Sodium chloride, SDS and phenylmethylsulphonylfluoride (PMSF) were purchased from Sigma Chemical Co. (St. Louis, MO, USA). Tris(hydroxymethyl)-aminomethane and glycine were purchased from USB corporation (Cleveland, OH, USA). Leupepth and pepstatin were from Roche Diagnostics GmbH (Mannheim, Germany). Acrylamide was purchased from Bio Rad Laboratories (Hercules, CA, CA) and nitrocellulose membrane from Bioexpress (Kaysville, UT, USA). Primary antibody against cleaved caspase-3 was purchased from Cell Signaling Technology, Inc., (Beverly, MA, USA) and primary antibodies against caspase-8, caspase-9 and β-actin were obtained from Santa Cruz Biotechnology, Inc. (Santa Cruz, CA, USA). Horseradish peroxidase conjugated goat anti-rabbit and anti-mouse secondary antibodies were purchased from BD Biosciences (Rockville, MD, USA). ECL Kit was bought from Amersham Biosciences (Piscataway, NJ, USA). TACS TdT *in situ* apoptosis detection kit was from R&D systems (Minneapolis, MN, USA). Other reagents were obtained in their highest purity grade available commercially.

### Synthesis of SD

Sarcophine was isolated from the soft coral *Sarcophyton glaucum* by multiple extractions with petroleum ether at room temperature as reported [14, 20] at the laboratories of Faculty of Pharmacy, Misr International University, Cairo, Egypt. The dried extract was evaporated under reduced pressure and chromatographed on silica gel column using hexane: ethyl acetate (1:2) as eluent. Pure sarcophine was obtained by crystallization from ethanol. SD was synthesized according to the following procedure: sarcophine was reduced to its lactone opened ring analog (1 mmol) to which selenium dioxide (98%, 1 mmol) in dry 1,4-dioxane (30 mL) was added and the reaction mixture was stirred at room temperature for 15 min and followed by TLC to check for completion of reaction. Water was then added to the reaction mixture, and the product was extracted with CH_2_Cl_2_. Saturated NaHCO_3_ solution was used to wash the CH_2_Cl_2_ layer which was dried over anhydrous Na_2_SO_4_. The solvent was evaporated and the residue was chromatographed on silica gel using hexane: ethyl acetate (1:2) as an eluent to obtain SD (90% yield) [14].

The structure of SD was fully characterized as shown in Figure 1 by spectroscopic methods and was identical to analytical sample prepared according to previous reported method of synthesis [14, 20]. Purity was confirmed by HPLC.

### Animals

Female SKH-1 mice were purchased from Charles River Laboratories (Wilmington, MA, USA). All mice were housed in the College of Pharmacy animal facility under climate-controlled environment with a 12 hours light/dark cycle. Mice were allowed free access to food pellets and water placed inside the food chamber on top of the cage cover. The experimental protocol was approved by the Institutional Animal Care and Use Committee.

### UVB source

The UVB irradiation unit, manufactured by Daavlin Corporation (Bryan, OH, USA) consists of four UVB lamps. The exposure dose can be controlled by using two Daavlin flex control integrating dosimeters. The dose of UVB exposure is expressed in millijoules/cm^2^ (mJ/cm^2^).

### UVB-initiated and UVB-promoted skin tumor development protocol

The tumorigenesis protocol as described by Dwivedi *et al.* [21] was used. Female SKH-1 mice were randomly divided into two groups having 27 mice per group, control and SD treatment. Both initiation as well as promotion was induced by UVB radiation (180 mJ/cm^2^). During the initiation phase, control group was treated with 100 μL of acetone and SD treatment group was treated with 100 μL of SD (30 μg/100 μL of acetone) 1 h prior to UVB exposure. The treatment and UVB exposure were done every day at the same time and was continued for 14 days. During the promotion phase, both control and SD treatment groups were treated in the same way as they were treated during the initiation phase. However, the treatment was done only twice a week (Tuesday and Friday) and was continued until the next 30 weeks for the promotion phase. Tumor counts and group weights were taken once every week.

### Effects of SD on tumor area of SKH-1 mice

Pictures of the mice were taken at the end of tumorigenesis protocol (the 30^th^ week of UVB-promotion). These digital photographs of the mice were used to calculate the visual surface area for each mouse and the tumor associated with each mouse. The boundaries of each tumor was clarified using Photoshop CS3 (Adobe Systems, San Jose, CA, USA), and the areas were determined with the measure-area feature of Image-Pro Plus 5.1 (Media Cybernetics, Inc., Bethesda, MD, USA).

### Histopathological analysis of mouse tumors

Mice were sacrificed by cervical dislocation at the termination of above protocol. Skin collected from mice was prepared for histopathological examination by immersion fixation in 10 % neutral buffered formalin for several days at room temperature. Fixed tissues were processed for paraffin-wax embedding, sectioned 4 to 6 micrometers thick, stained with hematoxylin and eosin (HE), and evaluated under light microscope.

### Tumorigenesis protocol for mechanistic studies

Female SKH-1 mice were randomly divided into two groups, control and SD treatment. Both initiation as well as promotion was induced by UVB radiation. During the initiation phase, control group was treated with 100 μL of acetone and SD treatment was treated with 100 μL of SD (30 μg/100 μL of acetone) 1 h prior to UVB treatment (180 mJ/cm^2^). This was done every day and was continued for seven days. One week after the initiation phase, promotion phase was started. During the promotion phase, control was treated with 100 μl of acetone and SD treated with 100 μL of SD (30 μg/100 μL of acetone) 1 h prior to UVB treatment (100 mJ/cm^2^) twice a week (Tuesday and Friday) for 19 weeks.

### Preparation of cell lysate for Western blotting

Mice from the mechanistic study protocol described above were sacrificed by cervical dislocation and epidermis of mice from both control and SD treated groups was collected. Cell lysate for Western blotting and analysis of caspase-3, -8, and -9 expressions were prepared as described earlier by Zhang *et al.* and Kundoor *et al.* [16–18]. Briefly, the fat and tumors in the skin of these two groups were removed and then epidermis was homogenized in 0.1 mM Tris-HCl (pH 7.4) containing 0.15 M sodium chloride. The epidermal homogenate was filtered by cheesecloth and then filtrate was centrifuged at 10,000 g for 20 min in the Beckman J2-21 Centrifuge. This pellet combined with 5% SDS containing 100 mM PMSF, 0.5% leupepth and 0.5% pepstatin was allowed to pass through 25G needle, centrifuged at 13,000 g for 20 min and heated in heating block (100 °C) for 5 min. Finally proteins were collected and used for determining expressions of caspase-3, -8, and -9 by SDS-PAGE and Western blotting.

### Western blot analysis of caspase -3, -8, and -9 expressions

Protein concentration was measured in each cell lysate by the protein assay (Pierce, Illinois) with albumin as a standard. Equal amount of protein lysates (60 μg) were resolved on SDS-poly-acrylamide gels and transferred to nitrocellulose membrane. Membranes were blocked for 1 h in 5% skim milk in TBS (10 mM Tris, 100 mM NaCl), and then probed with primary antibodies against caspase-3, -8, and -9. The secondary antibodies conjugated to horseradish peroxidase were used for development with the enhanced chemiluminescence detection (ECL) kit. Western blots were quantified using a UVP Biochem Gel Documentation system (UVP, Inc., Upland, California). To ensure equal protein loading, each membrane was stripped and reprobed with anti-β-actin antibody.

### Determination of DNA fragmented cells on skin sections

To identify DNA fragmentation of cells on the skin samples of mice from mechanistic study procedure, TACS TdT *in situ* apoptosis detection kit was used according to the procedure of manufacturer (R&D systems, Minneapolis, MN, USA). Briefly, skin samples were first fixed to prevent the loss of low molecular weight DNA fragments. To make the DNA accessible to the labeling enzyme, the cell membranes are permeabilized with proteinase K reagent. Endogenous peroxidase activity was quenched using hydrogen peroxide. Next, biotinylated nucleotides were incorporated into the 3′-OH ends of the DNA fragments by terminal deoxynucleotidyl transferase (TdT). The biotinylated nucleotides were detected by using streptavidin-horseradish peroxidase conjugate followed by the substrate, diaminobenzidine (DAB). DAB-stained samples were examined using a light microscope. The enzyme reaction generated an insoluble colored precipitate where DNA fragmentation occurred.

### Statistical analysis

The software INSTAT (Graph Pad, San Diego, CA, USA) was used to analyze the data. Chi Square was used for analyzing the data on tumor incidence. Student t-test was applied to compare the tumor multiplicity, weight gain, percent of tumor area, caspase-3, and caspase-8 expressions. Significance in all the cases was considered at *P* < 0.05.

## 5. Conclusions

The results from present study suggest that SD has modest chemopreventive effects at 30 μg per application and has an excellent potential to be a potent chemopreventive agent at higher concentrations for the non-melanoma skin cancer development by inducing apoptosis through extrinsic pathway. Future studies on dose-response, time-response, and pre-and post-treatment are needed to fully explore the skin cancer preventive effects of SD.
